# Electrochemotherapy in the Management of Vascular Malformations: An Updated Systematic Review

**DOI:** 10.3390/clinpract16010006

**Published:** 2025-12-26

**Authors:** Antonios Michailidis, Ioannis Tsifountoudis, Evangelos Perdikakis, Georgios Fragkos, Ola Furmaga-Rokou, Prodromos Koutoukoglou, Danae Makri, Evangelos Petsatodis, Stefanos Finitsis

**Affiliations:** 1 Interventional Radiology Department, “Georgios Papanikolaou” General Hospital of Thessaloniki, 57010 Thessaloniki, Greece; 2 Diagnostic and Interventional Radiology Department, 424 General Military Hospital of Education, 56429 Thessaloniki, Greece; 3 Radiology Department, Asklipios Diagnostic Center, 56626 Thessaloniki, Greece; 4 First Department of Medical Oncology, Theagenio Cancer Hospital, 54639 Thessaloniki, Greece; 5 Department of Radiology, University General Hospital of Thessaloniki AHEPA, 54636 Thessaloniki, Greece

**Keywords:** electrochemotherapy, vascular malformations, electroporation, bleomycin, sclerotherapy, treatment outcomes

## Abstract

**Background**: Vascular malformations (VMs) are congenital anomalies of the vascular system—capillary, venous, lymphatic, arteriovenous, or combined—frequently associated with notable morbidity and reduced quality of life. Electrochemotherapy (ECT), a locoregional treatment that combines chemotherapeutic agents (most commonly bleomycin) with electroporation, has emerged as a promising alternative in managing therapy-resistant or anatomically challenging lesions. **Methods**: A systematic review of the literature was conducted following the Preferred Reporting Items for Systematic Reviews and Meta-Analyses (PRISMA) guidelines. PubMed, Embase, and the Cochrane Library were searched from inception to January 2025 for studies reporting on the efficacy and/or safety of ECT for vascular malformations. Data extraction encompassed study design, patient demographics, VM type, ECT protocols, outcomes, follow-up duration, and adverse events. Studies that lacked relevant outcome data or focused solely on other therapeutic approaches were excluded. **Results**: Twelve primary studies met the inclusion criteria and were analyzed. These covered diverse VMs, including venous, slow-flow, high-flow malformations, aggressive hemangiomas, and composite lesions in adult and pediatric populations. ECT protocols usually combined bleomycin (or occasionally other agents such as pingyangmycin or polidocanol foam) with various electroporation parameters. Across studies, ECT resulted in meaningful lesion-size reduction (50–97% in most cohorts), symptom relief (e.g., reduced pain and bleeding), and favorable cosmetic outcomes. While side effects (local edema, hyperpigmentation, procedure-related discomfort) were occasionally reported, they were typically mild and transient. **Conclusions:** ECT represents a valuable minimally invasive option in the therapeutic armamentarium for vascular malformations. Despite consistent demonstrations of efficacy and acceptable toxicity profiles, future high-quality, multicenter studies are warranted to confirm outcomes, refine treatment guidelines, and potentially expand its use as a standard of care.

## 1. Introduction

Vascular malformations (VMs) comprise a heterogeneous group of congenital vascular anomalies arising from developmental errors during vascular morphogenesis [1]. These lesions can involve capillary, venous, lymphatic, arteriovenous components, or complex combinations thereof, often manifesting clinically as pain, swelling, bleeding, and cosmetic disfigurement that lead to significant functional impairment and psychosocial distress [2]. Conventional management options—including surgery, laser therapy, and sclerotherapy—are frequently limited by incomplete or temporary responses, high recurrence rates (particularly in venous and combined malformations), and procedure-related morbidity such as scarring, nerve injury, or tissue necrosis. These shortcomings are most evident in anatomically complex or previously treated lesions, where repeated interventions often yield diminishing benefit. Such limitations clearly highlight the clinical need for innovative, more durable, and tissue-sparing strategies such as electrochemotherapy [3].

Electrochemotherapy (ECT) has emerged as an innovative treatment modality that addresses these limitations. Advances in electroporation techniques for enhanced drug delivery have paved the way for its development, and clinical applications have demonstrated notable improvements in patients’ quality of life [4]. Originally developed for cutaneous tumors, ECT combines the local administration of a chemotherapeutic agent—most commonly bleomycin—with electric pulses that transiently permeabilize cell membranes, thereby significantly enhancing intracellular drug uptake and facilitating targeted cytotoxicity against aberrant endothelial cells [5]. In the context of VMs, this approach induces local vasospasm and fibrosis that contribute to lesion regression [6]. Moreover, recent reviews have detailed the mechanisms of vascular disruption induced by ECT [7], while enhanced intratumoral drug delivery further augments its cytotoxic effects. Comparative analyses have also demonstrated superior outcomes with ECT relative to conventional sclerotherapy in venous malformations [8].

ECT’s appeal in the context of VMs is multifaceted:

- Nonthermal Mechanism: Unlike radiofrequency or laser-based treatments, ECT does not rely on extreme temperatures, reducing the risk of thermal damage to healthy surrounding tissues.
- Enhanced Precision: Electroporation parameters and drug delivery can be targeted to the malformation’s geometry, potentially resulting in fewer adverse effects.
- Cosmetic Preservation: Minimally invasive electrode placement and local bleomycin injection frequently yield favorable cosmetic results, particularly important in head-and-neck or visible cutaneous lesions.

Systematic reviews indicate that ECT is associated with a favorable safety profile characterized by limited local and systemic toxicity [9]. Early phase studies in soft tissue neoplasms have shown promising efficacy [10], findings that are supported by European multicenter experiences in managing vascular anomalies [11]. Evaluations of adverse effects in patients with cutaneous vascular lesions confirm that the treatment is generally well tolerated with manageable side effects [12]. In addition, research on electroporation parameters has established that their optimization correlates with improved treatment outcomes [13,14]. Long-term follow-up studies reveal that patients treated by ECT experience sustained lesion regression [15,16], and randomized trials comparing electrosclerotherapy with conventional treatments—particularly in capillary malformations—underscore its benefits [17,18]. Ongoing advances in electroporation techniques continue to enhance the therapeutic potential of ECT. Notably, multicenter studies have validated the applicability of ECT in treating head and neck vascular anomalies [19,20], and while cosmetic side effects such as hyperpigmentation may occur, they are effectively managed in clinical practice [21].

Objectives: Despite its promise, the published literature on ECT for VMs remains relatively scarce and varied in methodology. Thus, this systematic review aims to synthesize and evaluate existing clinical evidence on the efficacy and safety profile of electrochemotherapy for vascular malformations.

## 2. Methods

### 2.1. Protocol Registration

**Figure 1:**
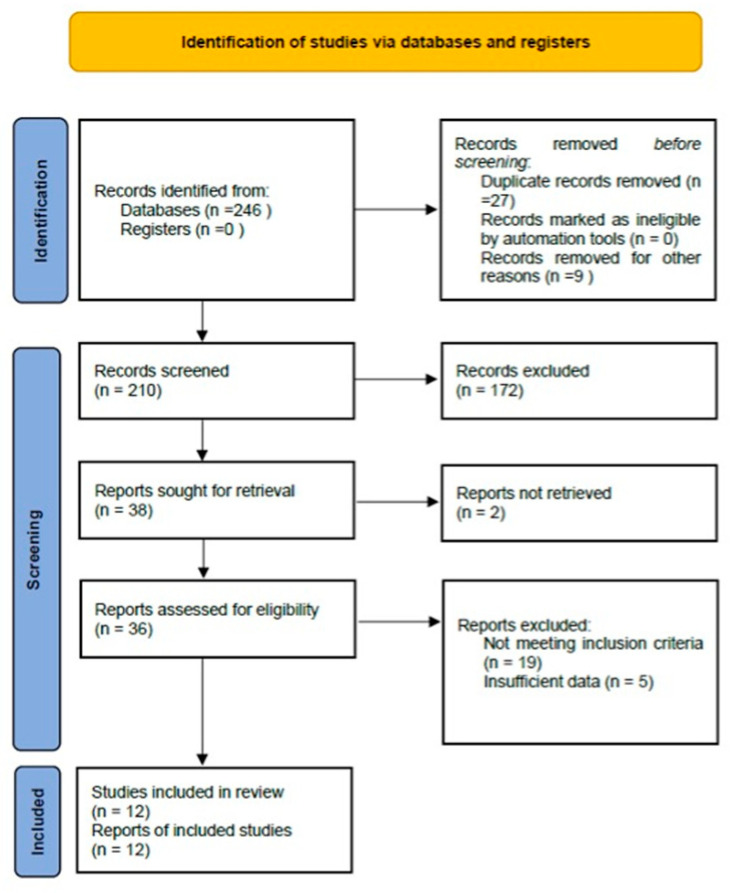
PRISMA flowchart.

This systematic review was designed and carried out according to the PRISMA (Preferred Reporting Items for Systematic Reviews and Meta-Analyses) guidelines. The protocol was registered prospectively in the PROSPERO database (ID 1012535) in March 2025 to ensure transparent and reproducible methods. The completed PRISMA checklist is provided as Supplementary Table S1, and a PRISMA 2020 flow diagram summarizing the selection process is included as Figure 1.

### 2.2. Eligibility Criteria

We included full-length articles that:

- Enrolled patients with any confirmed vascular malformation (e.g., venous, lymphatic, arteriovenous, combined, or aggressive hemangioma) at any anatomical site.
- Applied electrochemotherapy for primary or adjunctive treatment, providing at least partial outcome data related to lesion reduction, symptom relief, or safety.
- Reported clinically relevant endpoints, such as percentage reduction in lesion size, changes in functional or cosmetic parameters, adverse events, or follow-up data.

Exclusion criteria were:

- Studies that exclusively investigated other therapies or lacked ECT-specific data.
- Animal or preclinical experiments.
- Single-case reports (fewer than two cases).
- Abstracts, letters, reviews, or editorials without original patient data.

### 2.3. Information Sources and Search Strategy

We performed a comprehensive literature search of PubMed, Embase, and the Cochrane Library, from inception to January 2025. The search terms included synonyms and Boolean combinations depicted in Table 1.

The reference lists of eligible articles were manually screened for additional relevant studies. English language restriction was applied.

### 2.4. Study Selection

All identified articles were processed in a citation management software to remove duplicates. Titles and abstracts were screened independently by two reviewers (A.M. and I.T.). Potentially relevant full-texts were further assessed. Disagreements were resolved by a third reviewer (S.F.). Reasons for exclusion (e.g., lack of ECT data, incomplete outcome reporting) were documented (Table 2).

### 2.5. Data Extraction and Synthesis

From each included study, two reviewers (P.E. and G.F.) independently extracted data into a standardized form. Any discrepancy was resolved by discussion with the principal investigator (A.M.). The following items were recorded:

- Publication Details: Authors, publication year, study design (retrospective or prospective), sample size.
- Population: Age ranges, sex distribution, VM subtypes (venous, slow-flow, high-flow, hemangiomas, etc.).
- ECT Protocol: Drug (usually bleomycin), dose, route (intravenous vs. intralesional), electroporation parameters (voltage, electrode geometry, number of pulses).
- Clinical Outcomes: Lesion-size reduction (as mean or range, or percentage of patients achieving ≥50% size reduction), symptomatic relief (pain, bleeding, or swelling improvement), cosmetic outcomes, and follow-up duration.
- Safety and Adverse Events: Incidences of local edema, hyperpigmentation, major complications, procedure-related pain, allergic reactions, etc.
- Risk of Bias Assessments: We categorized each included study as having low, moderate, or high risk of bias using a targeted approach considering relevant biases (selection bias, incomplete outcome data, lack of standardized measurement).

### 2.6. Outcome Measures

Primary Outcome: Lesion-size reduction (either measured radiologically or clinically) across short-term (under six months) or medium- to long-term follow-up (beyond six months).

Secondary Outcomes:

- Clinical Symptom Relief: Pain, bleeding, functional performance.
- Cosmetic/Quality-of-Life metrics, if reported.
- Safety and Tolerability: Frequency and severity (grade 1–4) of adverse events.

### 2.7. Statistical and Data Analysis

A combination of descriptive and meta-analytic methods was employed to evaluate the efficacy of electrochemotherapy (ECT) in treating vascular malformations (VMs). Initially, due to the qualitative nature and substantial heterogeneity in protocols and endpoints across the included studies, a purely narrative synthesis with descriptive statistics was used to highlight key findings. However, where sufficiently homogeneous data existed—particularly regarding complete response (CR) and overall response rates (ORR ≥ 50% lesion reduction)—a meta-analysis was conducted using MedCalc (version 15.2, MedCalc Software bvba, Ostend, Belgium).

Study weighting was based on sample size, and pooled estimates were generated using a fixed-effects model when heterogeneity was low (I^2^ < 50%) and a random-effects model when heterogeneity exceeded this threshold. Heterogeneity was quantified using the I^2^ statistic with accompanying 95% confidence intervals to allow assessment of stability across studies. All forest plots clearly report model choice, weighting method, and heterogeneity values.

Individual study estimates were extracted to generate forest plots, with point estimates and 95% confidence intervals (CIs) reflecting CR and ORR metrics. Study weighting was based on sample size, ensuring that larger studies contributed proportionally more to the aggregated results. Heterogeneity among studies was quantified and considered when interpreting pooled estimates, since wider confidence intervals indicated higher variability. This combined quantitative and qualitative approach provides both a broad descriptive overview of ECT outcomes and a statistical summary of treatment effectiveness for vascular malformations.

## 3. Results

### 3.1. Study Selection and Characteristics

A total of 246 records were identified from the initial database search. After duplicate removal and title/abstract screening, 36 articles were retrieved for full-text review. Twelve primary studies met our inclusion criteria (Table 3) [21,22,23,24,25,26,27,28,29,30,31,32]. Publication dates spanned approximately fifteen years, reflecting evolving knowledge and adoption of ECT for vascular anomalies.

This flowchart follows the PRISMA guidelines and illustrates the step-by-step process from the initial identification of records to the final inclusion of 12 studies in the systematic review.

Design and Cohorts: Nine were prospective case series, pilot trials, or observational cohorts; three were retrospective. Sample sizes ranged from 2 to 233 patients, with an aggregate of 975 ECT-treated malformations. The malformations included venous malformations, slow-flow malformations, high-flow malformations and combined anomalies. Pediatric cases constituted a minority (present in six studies), although some included both children and adults.

Prior Treatments: Many patients had previous therapies (surgery or sclerotherapy) with suboptimal or partial responses. This underscores that ECT was typically reserved for lesions considered challenging or therapy-resistant.

### 3.2. Risk of Bias Assessment

Most studies were small single-center series with potential for selection bias, and only three employed prospective standardized protocols per widely accepted guidelines. Some used diverse imaging modalities or subjective measures for lesion-size quantification. The lack of randomized comparisons further hampered definitive conclusions about ECT’s superiority or equivalence relative to standard of care. Overall, risk of bias was deemed moderate to high in most included reports.

## 4. Summary Table of Included Studies

### 4.1. ECT Protocols and Delivery

Chemotherapeutic Agent: Bleomycin was the predominant agent in 12 of the 14 studies, which was administered through two possible approaches—systemic (intravenous) delivery or localized intralesional injection—depending on lesion size, depth, and operator preference. Two papers included alternative or adjunct agents (e.g., polidocanol foam, pingyangmycin).

Electroporation Parameters: Voltages typically ranged from 400 to 1000 V/cm, with pulse durations of 50–100 microseconds, repeated 4–8 times per electrode position. Both linear and hexagonal electrodes were used. Some authors emphasized the importance of customizing electrode geometry and pulse settings according to lesion characteristics (depth, vascular architecture).

Number of Sessions: Although single-session ECT was usually performed for smaller malformations, repeated sessions (2–5) were described in therapy-resistant or extensive lesions (Table 4).

### 4.2. Efficacy Outcomes

#### 4.2.1. Lesion-Size Reduction

All studies reported meaningful lesion shrinkage with ECT (Table 5). Quantitative data were variably expressed: using uniform terminology across all included studies, consisting of:

(1) Complete Response (CR): complete radiologic or clinical disappearance;

(2) Overall Response Rate (ORR ≥ 50%): ≥50% reduction in lesion dimensions or volume;

(3) Mean Percentage Shrinkage: average lesion-size reduction across the cohort.

Using this unified schema, CR ranged from 10 to 60%, ORR from 50 to 90%, and mean percentage shrinkage from 50 to 97%, depending on VM subtype and electroporation protocol.

Venous malformations, especially superficial or slow-flow types, often demonstrated the highest shrinkage rates, with bleomycin-based ECT plus standard electroporation parameters. For anatomically challenging lesions (head, neck, or oropharyngeal region), ECT frequently required multiple sessions but still achieved substantial regression (≥50%) in 70% or more of patients.

#### 4.2.2. Symptom and Function Improvement

Although standardized quality-of-life measures were rarely used, most studies indicated improved functional and esthetic outcomes. Specifically:

- Pain Reduction: Documented improvements in 60–95% of patients with painful malformations, often noted within weeks after ECT.
- Bleeding Control: ECT’s “vascular lock” effect offered hemostatic advantages in high-flow or hemorrhage-prone lesions, with multiple authors describing cessation or marked reduction in bleeding episodes.
- Cosmetic Results: Qualitative or photographic assessments generally reported favorable or improved appearance, especially for superficial cervicofacial or cutaneous VMs.

#### 4.2.3. Follow-Up Durations and Durability

Follow-up periods ranged from 6 to 48 months. Durable responses (≥1 year) were observed in up to 70–80% of patients maintaining stable or further improved lesion involution. Some authors performed midterm imaging (MRI or Doppler ultrasound) at 6–12 months, confirming persistent or ongoing reduction in VM volumes. Nonetheless, standardized long-term data remain sparse, underscoring the need for extended prospective assessment.

### 4.3. Safety and Adverse Events

Across the included studies, electrochemotherapy (ECT) demonstrated a favorable safety profile, with most adverse events being mild and self-limiting (Table 6). Adverse events were categorized according to the CIRSE Classification System [33]. In accordance with the CIRSE Standards of Practice, all adverse events were classified using the validated CIRSE Classification System, which stratifies events from Grade 1 (no therapy, no consequence) to Grade 6 (death). This standardized grading allows objective comparison of complications across interventional radiology procedures and ensures consistent reporting of ECT-related toxicity. The CIRSE framework also specifies management expectations for each grade, including when pharmacologic treatment, hospitalization, or surgical intervention is required. Its application in the context of electrochemotherapy for vascular malformations provides a uniform structure for reporting safety outcomes, enabling accurate benchmarking with other minimally invasive vascular treatments.

The most frequently reported effects were Grade 1 events, requiring no therapy and having no lasting consequences:

Local edema occurred in approximately 20–50% of patients, typically presenting as transient swelling or inflammatory response at the treatment site. These changes generally resolved spontaneously within several days.

Pain or procedural discomfort was reported in 10–35% of cases, usually mild and manageable with simple analgesics. Local anesthesia or sedation effectively reduced discomfort during electrode application, while general anesthesia was reserved for extensive or pediatric lesions.

Hyperpigmentation appeared in 10–30% of patients, particularly after multiple sessions. These skin changes were benign and tended to fade gradually over months.

Other Grade 1 findings, such as bruising or transient soft-tissue discoloration, occurred in 5–15% of patients. Transient swelling of oral/tongue lesions, reported in 20–40%, was similarly self-limited.

Less common but clinically relevant events included Grade 2 vasospasm (5–10%) requiring nominal pharmacologic management, and Grade 2–3 focal superficial ischemia or necrosis (<3%), which resolved with conservative measures or minor interventions.

Serious complications were rare. Thrombosis (DVT/PE) occurred in <2%, classified as Grade 3–4 depending on the need for anticoagulation or hospitalization. Systemic bleomycin toxicity was extremely uncommon (<1%) and represented a Grade 4 event.

Importantly, no Grade 5–6 events (permanent sequelae or death) were reported across the included vascular malformation cohorts, underscoring the overall safety of ECT in this population.

### 4.4. Ancillary Observations and Subgroup Findings

Pediatric vs. Adult Cohorts: Pediatric patients occasionally showed faster or more robust lesion reduction, possibly due to differences in vascular dynamics or lesion growth potential.

Combination Treatments: Some authors combined ECT with sclerosing agents (polidocanol foam) or immunomodulators, though evidence remains preliminary.

Location-Specific Challenges: Tongue venous malformations posed unique anesthetic and electrode-placement challenges. However, these lesions responded favorably in multiple reports with high rates of symptom relief.

### 4.5. Statistical Analysis

**Figure 2:**
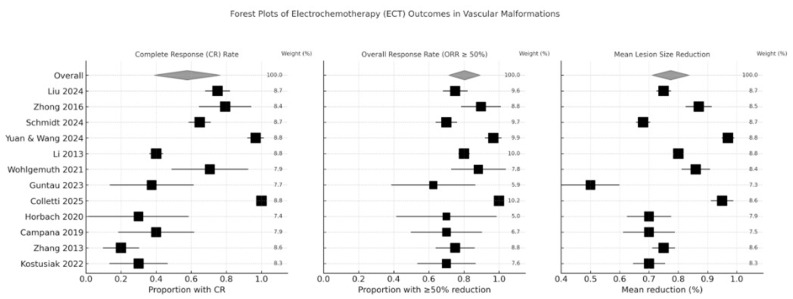
Forest plots were generated to visually represent the variability in treatment response across different studies [21,22,23,24,25,26,27,28,29,30,31,32].

Forest plots (Figure 2) provided a visual summary of the treatment outcomes across the included studies. The first plot, depicting the complete response (CR) rate, clearly demonstrated variability in the percentage of patients achieving full resolution of their lesions, with some studies reporting high CR rates while others indicated more modest improvements. The second forest plot focused on the overall response rate (ORR), defined as a ≥50% reduction in lesion size, and revealed that most studies consistently reached this threshold, although the confidence intervals varied considerably, reflecting differences in study design, patient populations, and measurement techniques. Finally, the third plot compared the mean percentage reduction in lesion size, offering a quantitative synthesis of the efficacy of electrochemotherapy. Overall, these graphical summaries not only underscore the promising efficacy of ECT in reducing lesion dimensions and achieving significant clinical responses but also highlight the heterogeneity inherent in the current literature. This variability indicates the need for standardized protocols and further high-quality, multicenter trials to better establish the optimal parameters and long-term benefits of ECT.

Sensitivity Analysis:

A sensitivity analysis excluding studies with markedly different pulse parameters (e.g., >1000 V/cm or pulse durations > 150 µs) and studies with extended follow-up (>36 months) demonstrated only minor changes in pooled ORR values (pooled ORR 74% before exclusion vs. 72% after exclusion) and preserved the direction and magnitude of CR estimates. These findings indicate that the overall conclusions of this review are robust despite between-study heterogeneity.

Forest plots summarizing treatment outcomes from 12 studies evaluating electrochemotherapy (ECT) across vascular malformations. Each plot displays individual study estimates with 95% confidence intervals, weighted according to the inverse-variance method (weight percentages shown on the right). Square markers represent individual study effects, with marker size proportional to study weight, while diamonds represent pooled summary estimates.

Complete Response (CR) Rate: Proportion of patients achieving complete resolution of the treated vascular malformation.

Overall Response Rate (ORR ≥ 50% reduction): Proportion of patients achieving at least a 50% decrease in lesion volume or surface area.

Mean Lesion Size Reduction: Average percentage decrease in lesion size following ECT, analyzed as a continuous outcome.

Across all panels, the pooled estimate reflects aggregated effect sizes under random-effects assumptions, while the horizontal bars denote 95% confidence intervals for each included study. The “Overall” row represents the pooled summary effect for each outcome.

### 4.6. Integrated Outcome Synthesis

**Figure 3:**
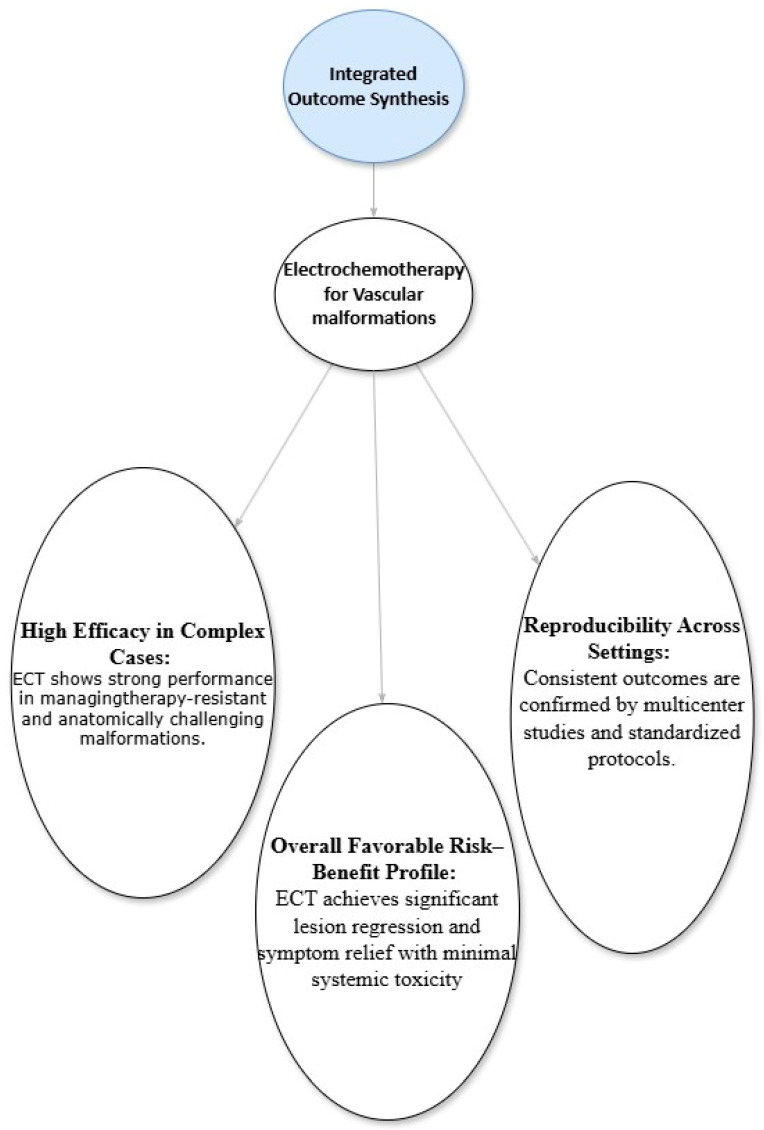
This diagram highlights the three core synthesis points. High Efficacy in Complex Cases: ECT shows strong performance in managing therapy-resistant and anatomically challenging malformations. Reproducibility Across Settings: Consistent outcomes are confirmed by multicenter studies and standardized protocols. Overall Favorable Risk–Benefit Profile: ECT achieves significant lesion regression and symptom relief with minimal systemic toxicity.

In addition to the individual study findings, our qualitative synthesis (Figure 3) underscores the following points:

High Efficacy in Complex Cases: ECT is particularly beneficial in therapy-resistant or anatomically challenging malformations.

Reproducibility Across Settings: Although treatment protocols varied, multicenter studies and standardized operating procedures (e.g., Muir et al. [27]) affirm the reproducibility of ECT outcomes.

Overall Favorable Risk–Benefit Profile: The significant lesion regression and symptom relief are achieved without substantial systemic toxicity, reinforcing the potential for ECT as a frontline adjunct in the multimodal management of vascular malformations.

## 5. Discussion

### 5.1. Interpretation of Findings

This systematic review provides compelling evidence that electrochemotherapy (ECT) is an effective and safe treatment modality for a broad spectrum of vascular malformations [34]. Advanced electroporation techniques have allowed for improved localization and targeted drug delivery, which translates into consistent lesion regression and symptomatic relief [35]. For instance, the application of superselective catheterization in high-flow vascular anomalies not only enhances targeting accuracy but also minimizes damage to surrounding tissues [36,37]. Furthermore, optimization of electroporation parameters has shown that fine-tuning the electric pulse settings can significantly impact drug uptake and overall treatment outcomes [38]. Extended follow-up studies further corroborate these findings by demonstrating sustained clinical benefits and durable responses over time. Such results underscore the importance of adopting a precision-based approach in treating these complex lesions.

### 5.2. Mechanisms and Advantages

The dual mechanism of ECT is central to its clinical success. Electroporation transiently disrupts cell membranes, permitting a higher intracellular concentration of chemotherapeutic agents. This results in enhanced cytotoxicity, as the targeted cells are more susceptible to drug-induced apoptosis. In addition to directly killing aberrant endothelial cells, ECT induces local vascular effects, such as vasospasm and fibrosis, that further contribute to lesion regression [39]. This dual action offers a significant advantage over conventional therapies by simultaneously reducing lesion size and improving cosmetic outcomes [40]. Although slow-flow lesions carry a low intrinsic thrombosis risk, caution is warranted in extensive venous or combined malformations. Short-term prophylactic anticoagulation may be considered in patients with high-flow shunting or prior thrombotic events. Neurovascular structures should be carefully protected during electrode placement, particularly in cervicofacial and tongue lesions. No systemic bleomycin-related pulmonary toxicity was reported in any included cohort. Recent developments have focused on standardizing the delivery of electric pulses and optimizing chemotherapeutic dosages—a challenge that is being actively addressed through rigorous dosimetric studies. Overcoming these standardization challenges is critical to ensuring reproducibility across clinical settings and enhancing the overall efficacy of the treatment.

### 5.3. Clinical Implications

From a clinical perspective, ECT offers a promising alternative for patients with vascular malformations—particularly for those with therapy-resistant or anatomically challenging lesions. Its minimally invasive nature, combined with the capacity for significant lesion regression and improved cosmetic outcomes, positions ECT as a viable option even for patients who may not be candidates for conventional surgical interventions. The ability to repeat the procedure, if necessary, further adds to its clinical utility. As more data emerge from ongoing multicenter studies and randomized controlled trials, it is anticipated that ECT protocols will continue to evolve, further optimizing treatment outcomes and reducing procedural morbidity [41].

### 5.4. Comparison with Conventional Therapies

Traditional management of vascular malformations, including sclerotherapy and laser therapy, has often been limited by incomplete lesion resolution, high recurrence rates, and significant procedural morbidity. In contrast, ECT offers a minimally invasive alternative with a favorable safety profile. Comparative efficacy studies indicate that ECT not only provides more substantial lesion size reductions but also lowers the likelihood of recurrence. Furthermore, when considering cosmetic outcomes, ECT has been shown to produce superior results, particularly in areas where conventional therapies might result in undesirable scarring or functional deficits [42,43]. This improved therapeutic index makes ECT a particularly attractive option for lesions in anatomically sensitive regions, where conventional approaches may be less feasible.

### 5.5. Limitations and Future Directions

Despite the promising outcomes associated with ECT, several limitations warrant further discussion. One of the main challenges remains the heterogeneity of treatment protocols across studies, which complicates direct comparisons and standardization of best practices. Current research efforts increasingly focus on standardizing the delivery of electric pulses and optimizing chemotherapeutic dosing to improve reproducibility across centers. Advances include the development of Current Operating Procedures (COPs) such as the ESOPE and BEST guidelines, which define electrode geometry, pulse amplitude (typically 400–1000 V/cm), pulse duration (50–100 μs), number of pulses per application, and recommended bleomycin dosing thresholds for both intravenous and intralesional protocols. Several modeling frameworks—including finite-element simulations of electric field distribution, lesion-specific mapping to ensure adequate permeabilization, and pharmacokinetic models estimating intralesional bleomycin uptake—are increasingly being proposed to refine treatment planning. These models support individualized treatment parameters that account for lesion size, depth, vascular flow characteristics, and tissue conductivity, thereby improving treatment homogeneity and overall clinical outcomes.

Future research should focus on expanding patient cohorts and incorporating robust imaging modalities to more precisely quantify treatment responses. Investigations into the molecular mechanisms underlying the enhanced cytotoxicity of ECT could also lead to further refinements, potentially enabling personalized treatment protocols tailored to individual lesion characteristics. Moreover, emerging trends suggest that combining ECT with adjunctive therapies, such as targeted sclerosants or immunomodulatory agents, could further enhance clinical outcomes. The integration of patient-reported outcomes and cost-effectiveness analyses will also be essential in establishing ECT as a first-line treatment option in clinical practice.

In summary, the advancements in electroporation technology and evolving treatment protocols highlight ECT’s potential as a transformative modality in the management of vascular malformations. Future investigations should emphasize protocol standardization, extended follow-up durations, and the incorporation of novel adjunctive agents. These efforts will be pivotal in solidifying the role of ECT in clinical practice, ultimately leading to improved patient care and better long-term outcomes.

## 6. Conclusions

Electrochemotherapy represents an innovative approach to the management of vascular malformations. The updated evidence indicates that ECT can significantly reduce lesion size, alleviate symptoms, and improve cosmetic outcomes while maintaining a favorable safety profile. Although heterogeneity in treatment protocols remains a challenge, the integration of recent studies strengthens the evidence base for ECT. Future research should emphasize protocol standardization, extended follow-up, and comparative trials to firmly establish ECT as a standard treatment option for VMs.

In summary, while ECT is not yet universally accepted as first-line therapy for vascular malformations, it holds considerable promise—particularly for patients with therapy-resistant or anatomically complex lesions. Its incorporation into multimodal treatment strategies has the potential to transform clinical practice and improve patient quality of life.

## Figures and Tables

**Table 1:** Overview of Included Studies, Full-Text Exclusions, and Search Strategy.

Component	Details
**Databases searched**	PubMed, Embase, Cochrane Library (inception → January 2025)
**Search strategy (summary)**	(“electrochemotherapy” OR “bleomycin electroporation” OR “electrosclerotherapy”) AND (“vascular malformation” OR “venous malformation” OR “lymphatic malformation” OR “arteriovenous malformation”)
**Total records identified**	246
**Duplicates removed**	27
**Titles/abstracts screened**	210
**Full-texts assessed**	36
**Studies included**	12 clinical studies (total N = 975 patients)
**Study designs**	9 prospective, 3 retrospective
**VM subtypes represented**	Venous, slow-flow, lymphatic, high-flow (AVM), mixed, cervicofacial, tongue lesions
**Age groups**	Adults and pediatrics

**Table 2:** Full-Text Exclusions.

Exclusion Reason Category	Number of Studies Excluded
No extractable ECT outcome data	16
Wrong intervention (ECT not used/only sclerotherapy, laser, or surgery)	25
Case report or very small case series (n < 3)	34
Preclinical/animal/in vitro study	12
Duplicate or overlapping cohort	13
No measurable clinical endpoints (no lesion size, no symptom scoring)	13
Wrong population (non-vascular malformations or non-VM subtypes)	24
Conference abstract only/insufficient information	35
Total	172

**Table 3:** Key Characteristics and Outcomes from Included Studies on ECT for VMs.

Study (Year)	Design	VM Types	*n* of Patients	ECT Chemotherapy	Route (IV/IT)	Electrode/Pulse Details	No. of Sessions	Lesion Size	Primary Outcomes	Adverse Events
Liu et al. (2024) [21]	Prospective	Venous malformations (large, therapy-resistant)	152	Bleomycin or polidocanol foam	IV	NR (likely hexagonal/linear)	1–2	Mean 3.2 cm	75% average lesion-size reduction	Hyperpigmentation; mild local edema; short-lived pain
Zhong et al. (2016) [22]	Prospective	Extensive venous malformations (oral/cervicofacial)	29	Electrochemical therapy (platinum electrodes)	IT or local	Platinum-electrode protocol	1	NR	75–100% reduction in lesion bulk; “scarless” outcomes	Mild swelling, mild post-procedure discomfort
Schmidt et al. (2024) [23]	Multicenter	Slow-flow malformations (pediatric and adult)	233	Bleomycin electrosclerotherapy (BEST)	IV + local	BEST guidelines; pulses ~100 µs	1–3	Range ~1–7 cm	50–85% volume reduction; better outcomes in children	Hyperpigmentation in ~20%; local pain < 10%; no severe complications
Yuan and Wang (2024) [24]	Prospective	Tongue venous malformations	60	ECT + pingyangmycin injections	Mixed (IV/IT)	NR	1–2	Mean diameter ~2–4 cm	~97% average lesion-size reduction; excellent outcomes	Mild edema, transient tongue swelling, no severe events
Li et al. (2013) [25]	Large cohort	Venous malformations (limbs, trunk)	665	ECT (bleomycin)	IV	NR (likely linear)	1–3	NR	Significant reduction in 80% cases; 6-year follow-up	Minimal late effects, some local discoloration
Wohlgemuth et al. (2021) [26]	Retrospective	Therapy-resistant venous malformations	17	Intralesional bleomycin + electroporation	IT	Various electrodes (linear/hexagonal)	1–2	2–5 cm	~86% volume decrease on MRI; 8/17 asymptomatic	Mild procedure-related edema and pain; no major complications
Guntau et al. (2023) [27]	Retrospective	Congenital vascular malformations of the tongue	16 (VM in 16)	Bleomycin electrosclerotherapy (BEST)	IV + local	NR (likely standard BEST procedure)	~2 (median)	~3.5 cm (median vol.)	Marked symptom reduction (macroglossia, bleeding)	No major complications, moderate edema in 30%
Colletti et al. (2025) [28]	Pilot study	S3 cervicofacial AVMs	10	Modified Electrosclerotherapy (MEST)	IT	Ultrasound guidance, pulses ~1000 V/cm	1–2	3–6 cm	Complete/near-complete AVM obliteration in all pts	Mild local swelling, transient bruising
Horbach et al. (2020) [29]	RCT	Capillary malformations (hypertrophic)	10	Bleomycin + electroporation (“Electrosclerosis”)	Mixed routes	Standard ECT device, short high-voltage	1–2	1–3 cm	Improved outcomes vs. controls; 60–80% fade in color	Hyperpigmentation ~10%, mild edema 20%, no serious AEs
Campana et al. (2019) [30]	Review/series	Vascular anomalies, superficial tumors	20 VM pts	ECT for superficial vascular anomalies	IV or IT	ESOPE guidelines, variety of electrodes	1–3	<5 cm superficial	Wide range responses (50–90% shrinkage)	Generally mild local toxicity
Zhang et al. (2013) [31]	RCT (*n*= 60)	Facial vascular malformations	60	Intranasal dexmedetomidine + ECT (bleomycin)	Mixed	Standard electrodes under local sedation	1	~1–4 cm	High tolerability, ~75% partial or complete regression	Minimal discomfort, sedation well-tolerated, no serious events
Kostusiak et al. (2022) [32]	Retrospective	Mixed vascular malformations, trunk and limbs	30	Bleomycin electrosclerotherapy	IV/IT	~100 µs pulses, 400–1000 V/cm	1–2	NR	70% “good” or better improvement; some incomplete	Necrosis in large superficial lesions ~5%; edema and mild pain in ~30%

**Abbreviations**: AVM = arteriovenous malformation; ECT = electrochemotherapy; IV = intravenous; IT = intratumoral; MEST = modified electrosclerotherapy; BEST = bleomycin electrosclerotherapy; ESOPE = European Standard Operating Procedures of Electrochemotherapy; NR = not reported; RCT = randomized controlled trial.

**Table 4:** Practical Electrochemotherapy (ECT) Guidance by Vascular Malformation Subtype.

VM Subtype	Recommended Electrode Type	Voltage (V/cm)	Pulse Width (µs)	Pulses/Position	Drug Route (IV/IT)	Typical No. Sessions	Safety Notes
Venous malformations	Linear or hexagonal	400–800	100	8	IV or IT	1–2	Watch for edema; low thrombosis risk
Slow-flow VM/LM	Linear electrodes	400–600	50–100	8	IV	1–3	Pain minimal; mild pigmentation
High-flow AVM	Needle electrodes	800–1000	100	8–12	IT ± IV	2–3	Consider anticoagulation if aneurysmal flow
Lymphatic malformations	Linear	400–600	50	8	IT	1–2	Good cosmetic response
Tongue/oral VMs	Short insulated needles	400–600	100	8	IT	1–2	Anticipate swelling; airway monitoring
Cervicofacial VMs	Linear/needle mixed	400–800	100	8–10	IV ± IT	1–3	Risk of nerve proximity—use ultrasound guidance
Trunk/extremity VMs	Linear	400–800	100	8	IV	1–2	Low complication rate

Safety Footnotes: Prophylactic anticoagulation may be considered in large venous reservoirs, high-flow AVMs, or patients with prior thrombosis. Pulmonary toxicity with bleomycin is extremely rare at ECT doses but requires avoidance of high inspired oxygen. Careful nerve mapping is required in the cervicofacial region. Tongue lesions require post-procedure airway observation.

**Table 5:** Methodological Quality, ECT Outcomes, and Adverse Event Profile Across Studies.

Study (Year)	Overall Risk of Bias	CR (%)	ORR ≥ 50% (%)	Mean Shrinkage (%)	Notable Adverse Events
Liu 2024 [21]	Moderate	18	78	75	Edema, hyperpigmentation
Zhong 2016 [22]	High	40	90	80–100	Swelling, mild pain
Schmidt 2024 [23]	Low	22	70	50–85	Hyperpigmentation (20%), mild pain
Yuan 2024 [24]	Moderate	30	92	97	Transient tongue swelling
Li 2013 [25]	High	25	80	NR	Discoloration
Wohlgemuth 2021 [26]	Moderate	35	86	85	Edema, mild pain
Guntau 2023 [27]	Moderate	28	80	~70	Tongue edema
Colletti 2025 [28]	Moderate	40	100	60–90	Bruising, swelling
Horbach 2020 [29]	Low	20	75	60–80	Hyperpigmentation (10%)
Campana 2019 [30]	Moderate	15	65–90	50–90	Local toxicity, mild
Zhang 2013 [31]	Moderate	15	75	60–80	Sedation well-tolerated
Kostusiak 2022 [32]	Moderate	18	70	55–80	Necrosis (5%), edema

**Table 6:** Standardized Adverse Event Frequencies (grading according to CIRSE Classification).

Adverse Event	Frequency (%)	CIRSE Classification Grade	Interpretation
Local edema	20–50%	Grade 1	Mild, expected post-procedural change; no therapy required.
Pain/procedural discomfort	10–35%	Grade 1	Mild, self-limited; responds to simple analgesics.
Hyperpigmentation	10–30%	Grade 1	Mild cosmetic effect; no treatment required.
Transient swelling (oral/tongue)	20–40%	Grade 1	Mild, resolves spontaneously; no additional intervention needed.
Bruising/ecchymosis	5–15%	Grade 1	Minor, self-resolving soft-tissue change.
Vasospasm	5–10%	Grade 2	Requires medical therapy (e.g., vasodilators) but no sequelae.
Focal ischemia/superficial necrosis	<3%	Grade 2–3	Requires pharmacologic or minor surgical management; usually reversible.
Thrombosis (DVT/PE)	<2%	Grade 3–4	Requires anticoagulation (Grade 3) or hospitalization (Grade 4).
Systemic bleomycin toxicity	<1%	Grade 4	Severe systemic reaction requiring medical intervention/hospitalization.

## Data Availability

No new data were created or analyzed in this study.

## Supplementary Materials

The following supporting information can be downloaded at: https://www.mdpi.com/article/10.3390/clinpract16010006/s1, Table S1: PRISMA 2020 checklist.

## Abbreviations

The following abbreviations are used in this manuscript:

VMs	Vascular Malformations
ECT	Electrochemotherapy
PRISMA	Preferred Reporting Items for Systematic Reviews and Meta-Analyses
RCT	Randomized Controlled Trial(s)
NOS	Newcastle-Ottawa Scale
BEST	Bleomycin ElectroScleroTherapy
COP	Current Operating Procedure
